# Supplementary material for: Empirical methods for controlling false positives and estimating confidence in ChIP-Seq peaks
Source: BMC Bioinformatics. 2008 Dec 5;9:523. doi: 10.1186/1471-2105-9-523 (PMC2628906; doi:10.1186/1471-2105-9-523)
Supplement: Additional File 1 — A variety of html documents from the USeq web site detailing the available applications, their best usage, output file type descriptions, command line menus, etc. [file 1471-2105-9-523-S1.zip › USeqUserGuides/JavaDoc.zip!/JavaDoc/trans/tpmap/TPMapDuplicateFilter.html]

TPMapDuplicateFilter


|  |  |  |
| --- | --- | --- |
| **Overview** \| **Package** \| **Class** \| **Use** \| **Tree** \| **Deprecated** \| **Index** \| **Help** | |  |
| **PREV CLASS**   **NEXT CLASS** | **FRAMES**    **NO FRAMES**     **All Classes** |  |
| SUMMARY: NESTED \| FIELD \| CONSTR \| METHOD | DETAIL: FIELD \| CONSTR \| METHOD |  |


---


## trans.tpmap Class TPMapDuplicateFilter

```
java.lang.Object
  trans.tpmap.TPMapDuplicateFilter
```

---

``` public class TPMapDuplicateFilter extends java.lang.Object ```

For extracting duplicate oligo/ features from Affy's bpmap file, can be used as a
stand alone but better to use `TPMapProcessor`.
The TPMapDuplicateFilter first creates a hashMap of unique oligo sequences paired with an
ArrayList of chip coordinates where a particular oligo was spotted. In most cases,
each oligo was spotted only once. In some cases the same oligo was placed on the array
many times. This repeat information will be used by the CelMapper program to average
the intensities of all the repeats, for a particular oligo, and assign this value to
each spot. This repeat information is saved as an unordered array of TPMapFeature[] to disk.
The TPMapDuplicate Filter then reads line by line thru the bpmap txt file and removes any
adjacent repeated sequences. (After averaging they all will have the same value.)
It then removes any oligo that maps more than once to the genome. This "dupFree"
bpmap text file is written to disk.
Recommend rewriting this to use first hashMap to eliminate dups and skip the second step.

---

**Constructor Summary**

`TPMapDuplicateFilter(java.io.File bpmapFile,
java.io.File saveDirectory,
java.lang.String newFileName)`
  
          For running in combination with TPMapProcessor

`TPMapDuplicateFilter(java.lang.String fileName)`
  
          For running this alone.


| **Method Summary** | |
| --- | --- |
| `void` | `filterTPMap()` |
| `int` | `getLineNumber()` |
| `void` | `loadBPHashMap(int initialValue, java.io.File file)` |
| `static void` | `main(java.lang.String[] args)` |
| `static boolean` | `matches(TPMapFeature a, TPMapFeature b)`             Only checks coordinates! |
| `void` | `removeAdjacentDuplicates()`             Throws out data lines that are immediately adjacent and have the same sequence. |
| `void` | `removeDuplicateMappingOligos(java.io.File bpmap)`             Removes any oligo that is found more than once in the bpmap file. |

**Methods inherited from class java.lang.Object**

`equals, getClass, hashCode, notify, notifyAll, toString, wait, wait, wait`

**Constructor Detail**

### TPMapDuplicateFilter

```
public TPMapDuplicateFilter(java.lang.String fileName)
```

:   For running this alone.

---


### TPMapDuplicateFilter

```
public TPMapDuplicateFilter(java.io.File bpmapFile,
                            java.io.File saveDirectory,
                            java.lang.String newFileName)
```

:   For running in combination with TPMapProcessor


**Method Detail**

### filterTPMap

```
public void filterTPMap()
```

---


### removeDuplicateMappingOligos

```
public void removeDuplicateMappingOligos(java.io.File bpmap)
```

:   Removes any oligo that is found more than once in the bpmap file. Run
    removeAdjacentDuplicates() first!

---


### removeAdjacentDuplicates

```
public void removeAdjacentDuplicates()
```

:   Throws out data lines that are immediately adjacent and have the same sequence.

---


### loadBPHashMap

```
public void loadBPHashMap(int initialValue,
                          java.io.File file)
```

---


### matches

```
public static boolean matches(TPMapFeature a,
                              TPMapFeature b)
```

:   Only checks coordinates!

---


### main

```
public static void main(java.lang.String[] args)
```

---


### getLineNumber

```
public int getLineNumber()
```


---


|  |  |  |
| --- | --- | --- |
| **Overview** \| **Package** \| **Class** \| **Use** \| **Tree** \| **Deprecated** \| **Index** \| **Help** | |  |
| **PREV CLASS**   **NEXT CLASS** | **FRAMES**    **NO FRAMES**     **All Classes** |  |
| SUMMARY: NESTED \| FIELD \| CONSTR \| METHOD | DETAIL: FIELD \| CONSTR \| METHOD |  |


---
